# Supplementary material for: Thermo-amplifier circuit in probiotic E. coli for stringently temperature-controlled release of a novel antibiotic
Source: J Biol Eng. 2024 Nov 12;18:66. doi: 10.1186/s13036-024-00463-y (PMC11559228; doi:10.1186/s13036-024-00463-y)
Supplement: Supplementary file 1 — Additional file 1: Fig. S1. Comparative mCherry production by the pNOSO-mCherry and pUC-Tlp-mCherry plasmids in ClearColi BL21 DE3. Fig. S2. Comparative analysis of biomass and darobactin production by the pNOSO-darABCDE and pT7-DarA plasmids in ClearColi BL21 DE3. Fig. S3. Comparative analysis of darobactin production by the pNOSO-darABCDE and pTlp-DarA-AT plasmids in EcN-T7 and EcN strains respectively. Fig. S4. qPCR analysis-based assessment of pTAMP-DarA-AT recombinant plasmid retention in EcN with and without antibiotic supplementation. Fig. S5. Growth kinetics of the engineered strains at 37 °C and 40 °C. Fig. S6. darA gene expression level by the pTlp-DarA-AT plasmid. Fig. S7. Minimum Inhibitory Concentration (MIC) of darobactin for Pseudomonas aeruginosa PAO1 strain. Fig. S8 & Fig. S9. Sequence annotated maps of pTlp-DarA-AT and pTAMP-DarA-AT recombinant plasmids. Table S1. Nucleotide sequences of genetic modules. Table S2. Primer sequences for qRT-PCR and qPCR analysis. [file 13036_2024_463_MOESM1_ESM.docx]

**Supplementary Information**

**Thermo-amplifier circuit in probiotic *E. coli* for stringently temperature-controlled release of a novel antibiotic**

Sourik Dey^1^, Carsten E. Seyfert^2,3^, Claudia Fink-Straube^1^, Andreas M. Kany^2,3^, Rolf Müller^2,3^, Shrikrishnan Sankaran*^1^

^1^ INM - Leibniz Institute for New Materials, Campus D2 2, 66123 Saarbrücken, Germany

^2^ Microbial Natural Products, Helmholtz Institute for Pharmaceutical Research Saarland (HIPS), Helmholtz Centre for Infection Research (HZI) and Department of Pharmacy at Saarland University, Campus Building E8.1, 66123 Saarbrücken (Germany)

^3^ German Centre for Infection Research (DZIF), partner site Hannover-Braunschweig, Germany

*E-mail: **shrikrishnan.sankaran@leibniz-inm.de**


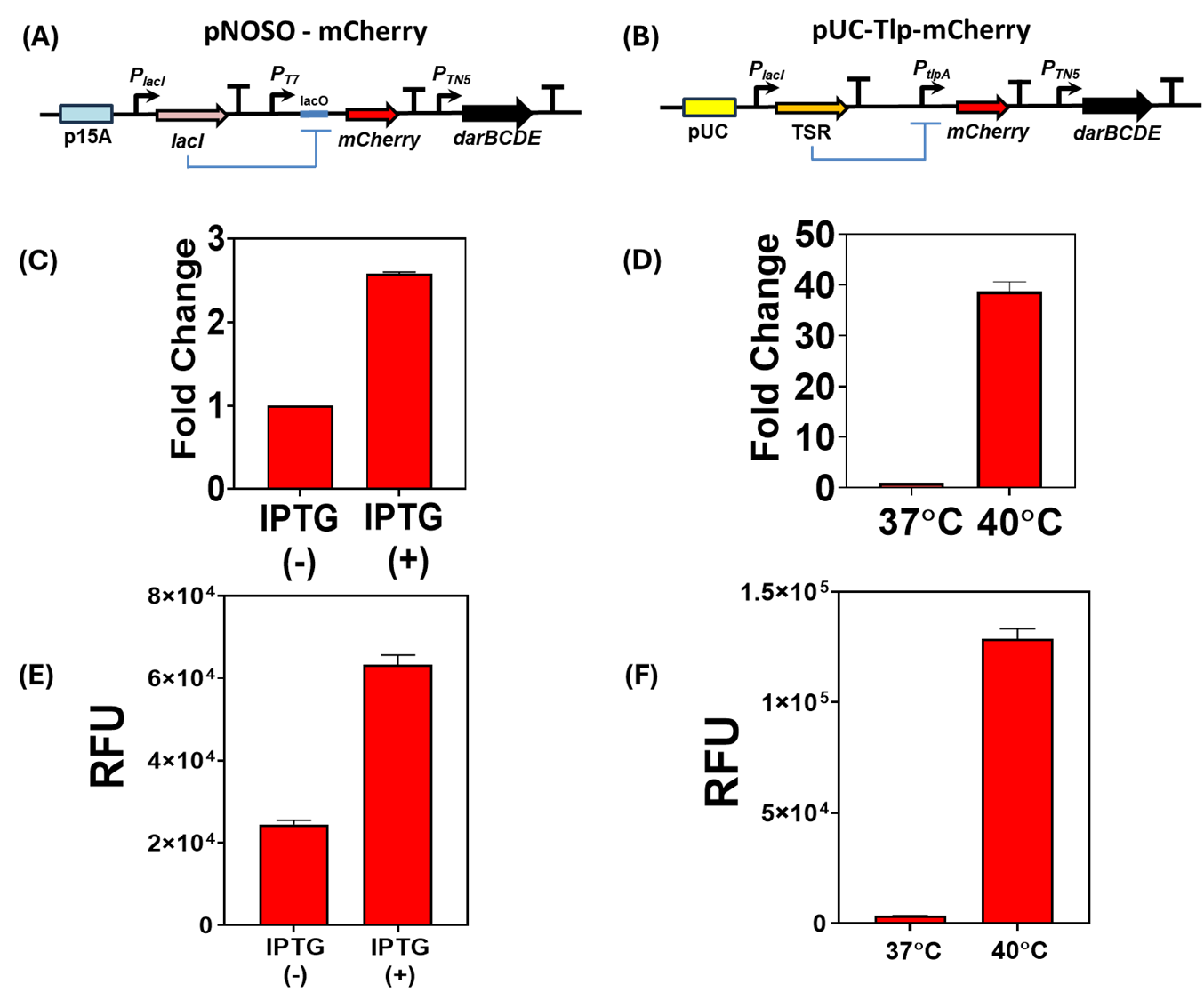


**Supplemental Figure 1.** (A) Schematic representation of the pNOSO-mCherry genetic circuit (B) Schematic representation of the pUC-Tlp-mCherry genetic circuit (C) Fold Change of P*_T7_* promoter driven mCherry expression in pNOSO-mCherry ClearColi strain after 24 h incubation at 37°C. The data represents the increase in mCherry expression post 500 µM IPTG induction [IPTG (+)] in comparison to the non-induced sample [IPTG (-)]. The error bars represent the standard deviation based on three independent measurements (D) Fold Change of P*_tlpA_* promoter driven mCherry expression in pUC-Tlp-mCherry ClearColi strain after 24 h incubation at 37°C and 40°C respectively. The data represents the increase in mCherry expression at 40°C incubation temperature in comparison to the 37°C incubated samples. The error bars represent the standard deviation based on three independent measurements (E) Relative Fluorescence Units (RFU) of mCherry produced by the pNOSO-mCherry ClearColi strain after 24 h incubation at 37°C. The data represents the increase in mCherry expression post 500 µM IPTG induction [IPTG (+)] in comparison to the non-induced sample [IPTG (-)]. The error bars represent the standard deviation based on three independent measurements (F) Relative Fluorescence Units (RFU) of mCherry produced by the pUC-Tlp-mCherry ClearColi strain after 24 h incubation at 37°C and 40°C respectively. The data represents the increase in mCherry expression when incubated at 40°C in comparison to 37°C. The error bars represent the standard deviation based on three independent measurements.


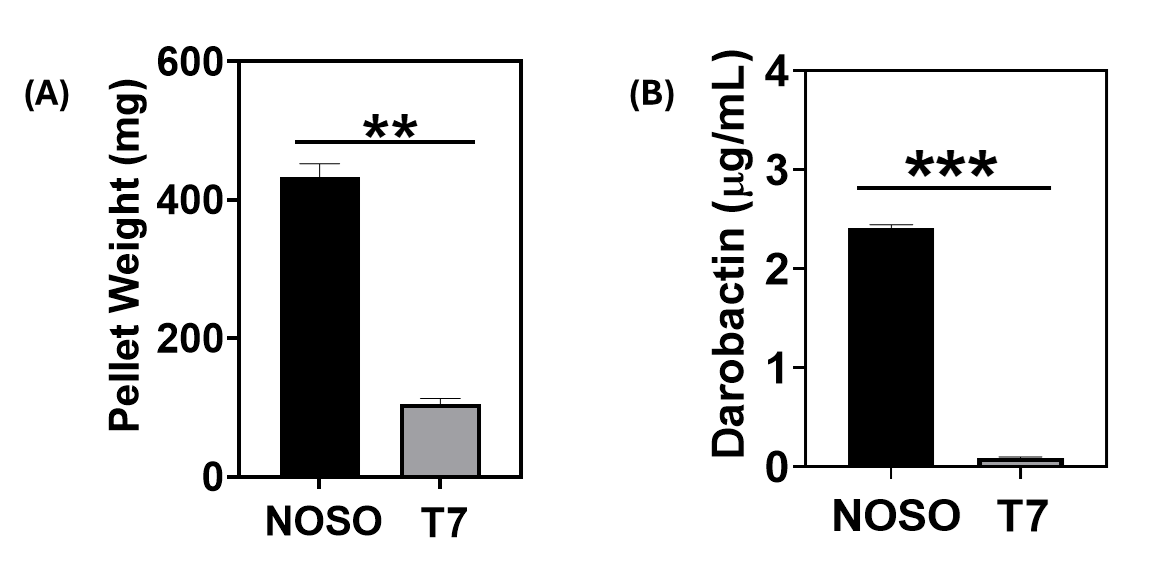


**Supplemental Figure 2.** (A) Biomass comparison in terms of the bacterial pellet wet weight [in milligrams (mg)] obtained from a 25 mL culture after 24 h incubation at 37°C. The pNOSO-darABCDE ClearColi strain was induced with 500 µM IPTG at log phase (OD_600_=0.4), whereas the pT7-DarA ClearColi strain was not subjected to IPTG supplementation. The error bars represent standard deviation based on three independent measurements (**p *<* 0.001 as calculated by paired t-test) (B) Darobactin concentration (in µg/mL) in the liquid medium of IPTG-induced pNOSO-darABCDE ClearColi strain in comparison to the non-IPTG supplemented pT7-DarA ClearColi strain after 24 h incubation at 37°C. The error bars represent standard deviation based on three independent measurements (***p *<* 0.0001 as calculated by paired t-test).


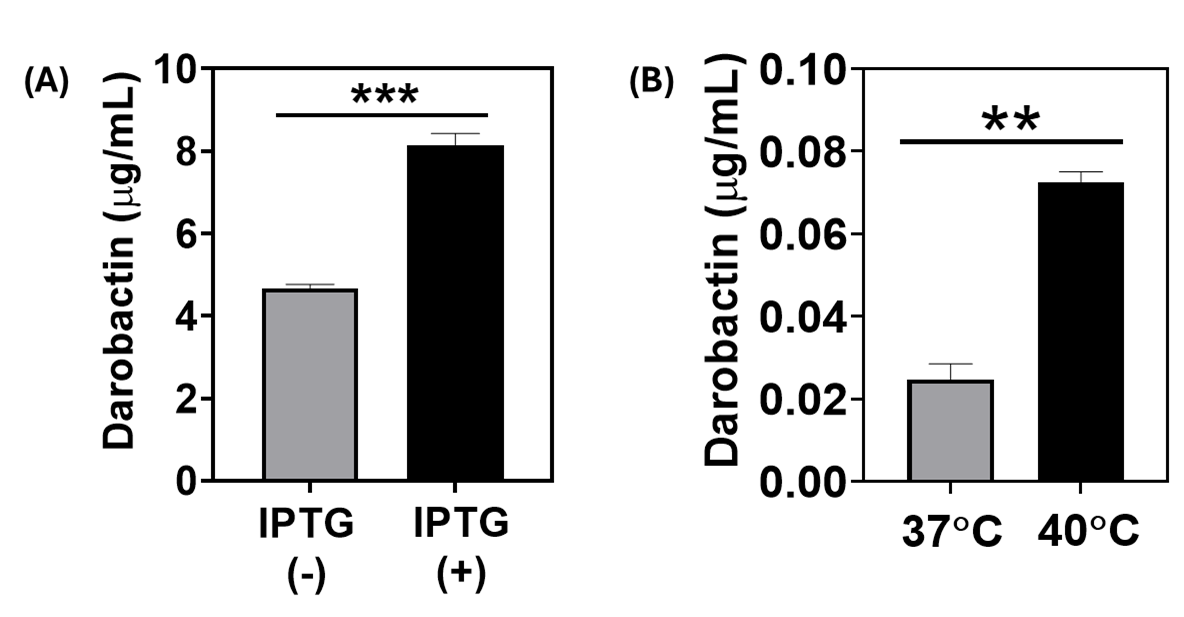


**Supplemental Figure 3.** (A) Darobactin concentration in the liquid medium (in µg/mL) of the pNOSO-darABCDE *E. coli* Nissle 1917 – T7 (EcN-T7) strain after 24 h of incubation at 37°C. For IPTG induction, the bacterial sample was grown till OD_600_ = 0.4 in Formulated Media (FM) supplemented with 50 µg/mL kanamycin and then induced with 500 µM of IPTG. The error bars represent standard deviation based on three independent measurements (***p = 0.001 as calculated by paired t-test) (B) Darobactin concentration in the liquid medium (in µg/mL) of the pTlp-DarA-AT *E. coli* Nissle 1917 (EcN) strain after 24 h incubation at 37°C and 40°C respectively (without antibiotic supplementation). The error bars represent standard deviation based on three independent measurements (**p = 0.0058 as calculated by paired t-test).


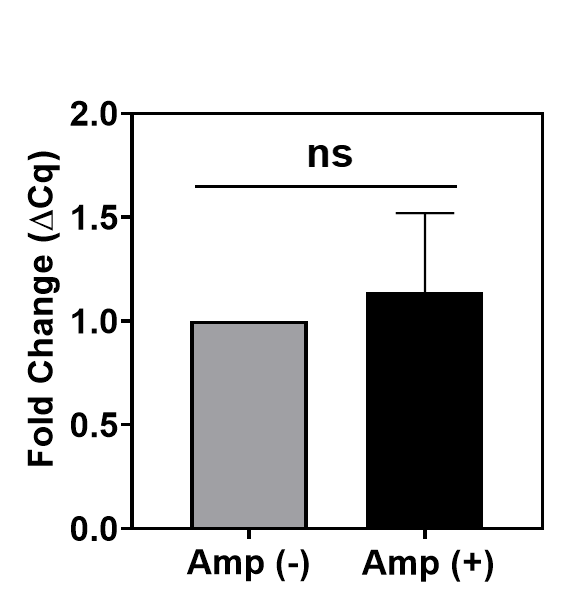


**Supplemental Figure 4.** qPCR analysis-based detection of the pTAMP-DarA-AT recombinant plasmid in EcN cultivated with and without ampicillin (100 µg/mL) supplementation at 40°C, compared over 10 and 50 generation numbers. The error bars represent standard deviation based on three independent measurements (^ns^p = 0.5955 as calculated by paired t-test).


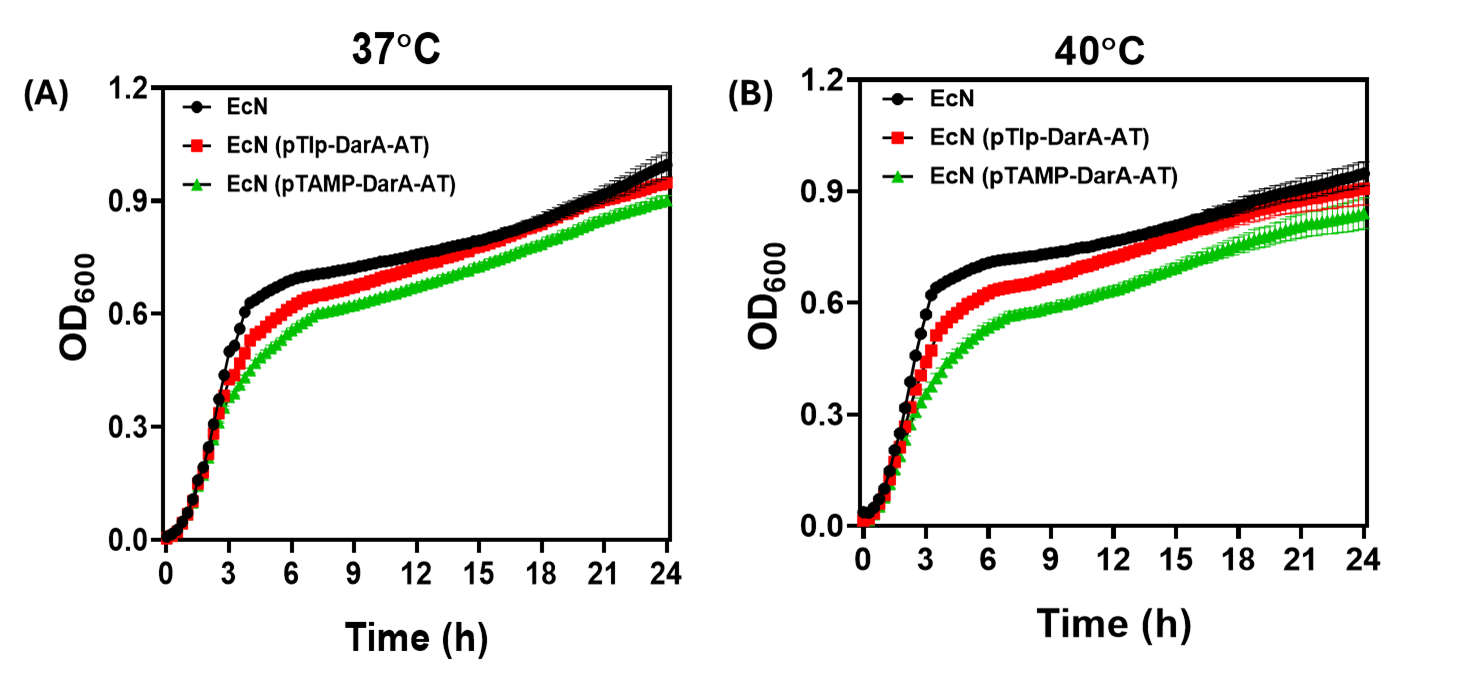
**Supplemental Figure 5.** (A) Growth Kinetics of Wild Type *E. coli* Nissle (EcN), thermo-responsive *E. coli* Nissle (pTlp-DarA-AT EcN) and thermo-amplifier *E. coli* Nissle (pTAMP-DarA-AT EcN) strains at 37°C for a 24 h incubation period. The samples were shaken continuously, and their Optical Density (OD_600_) measured at equal time intervals. The error bars represent standard deviation based on three independent measurements (B) Growth Kinetics of Wild Type *E. coli* Nissle (EcN), thermo-responsive *E. coli* Nissle (pTlp-DarA-AT EcN) and thermo-amplifier *E. coli* Nissle (pTAMP-DarA-AT EcN) strains at 40°C for a 24 h incubation period. The samples were shaken continuously, and their Optical Density (OD_600_) measured at equal time intervals. The error bars represent standard deviation based on three independent measurements.


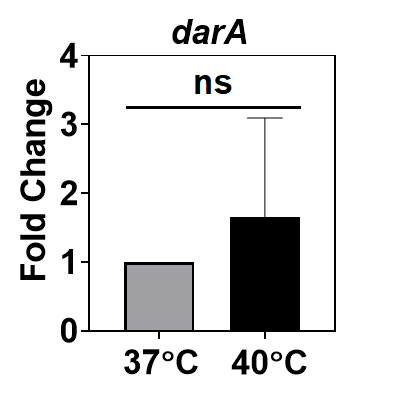


**Supplemental Figure 6.** Fold change in *darA* gene expression driven by the *P_tlpA_* promoter of pTlp-DarA-AT construct in EcN at 37°C and 40°C after 6 h incubation in FM (without antibiotic supplementation). The error bars represent standard deviation based on six independent measurements (^ns^p = 0.3137 as calculated by paired t-test).


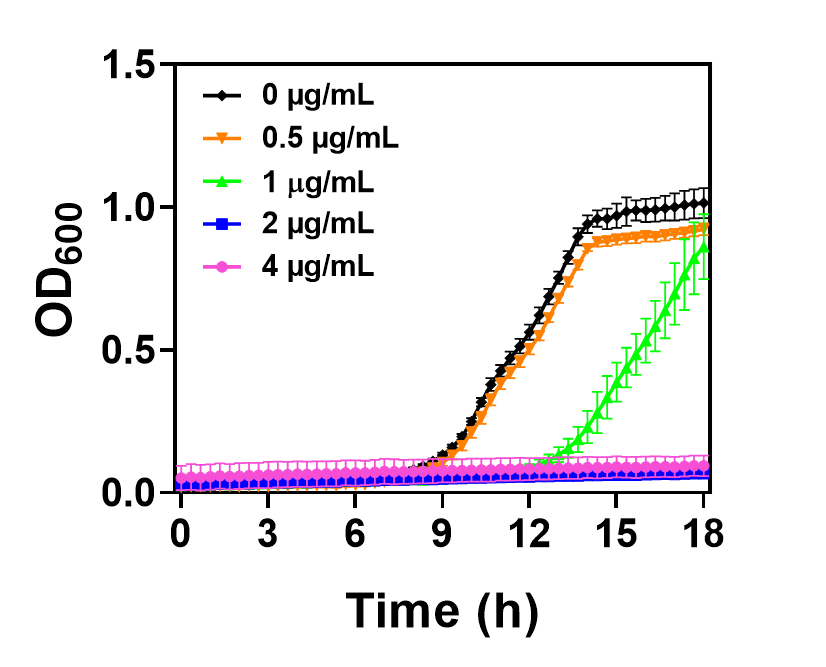


**Supplemental Figure 7.** Growth Kinetics of *Pseudomonas aeruginosa* PAO1 (DSMZ 22644) in Formulated Media (FM) supplemented with 0.5, 1, 2 and 4 µg/mL of darobactin respectively. Non-darobactin supplemented samples were used as a control (0 µg/mL). Absorbance at 600 nm (Optical Density or OD600) of the samples was recorded at regular intervals at 37°C incubation temperature along with continuous orbital shaking. The error bars represent standard deviation based on three independent measurements.


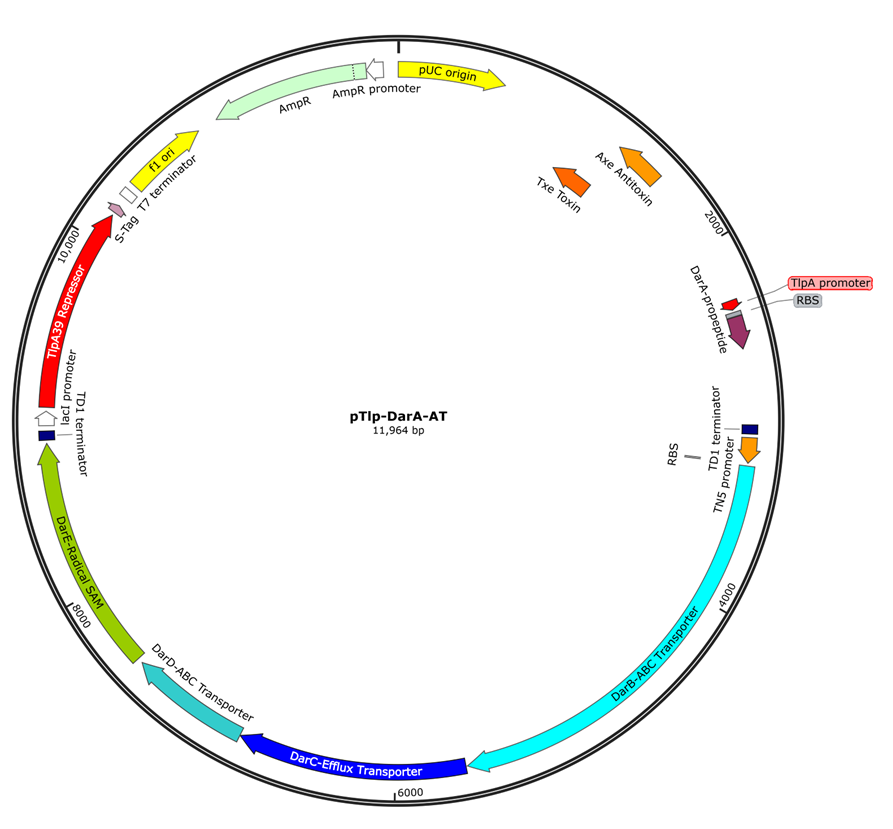


**Supplemental Figure 8.** Sequence annotated map of the pTlp-DarA-AT recombinant plasmid


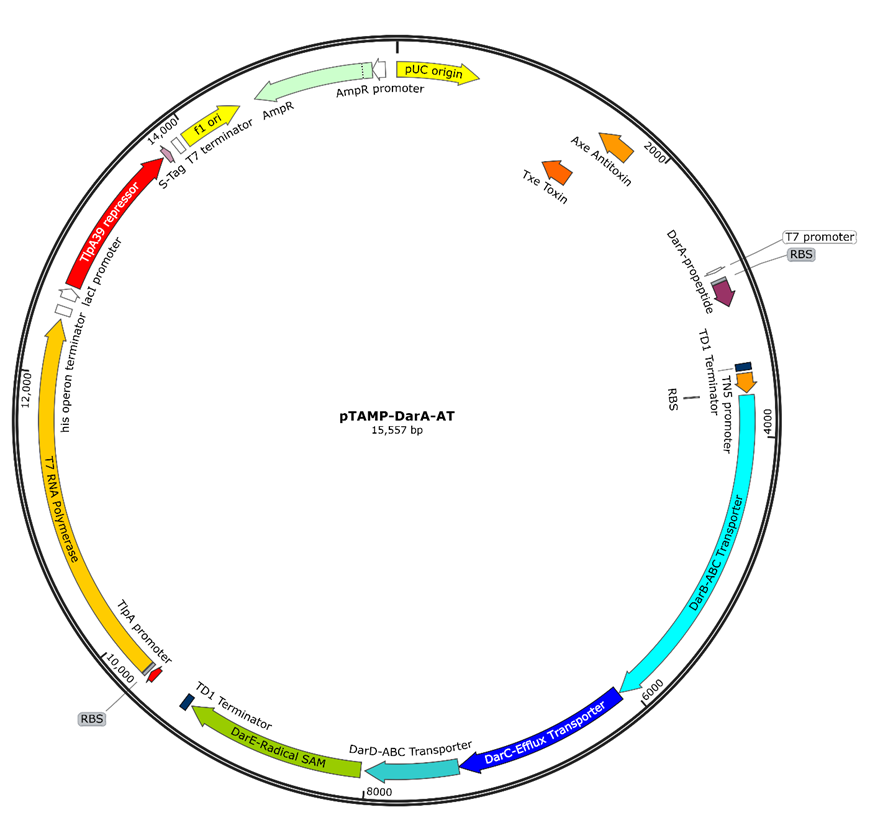


**Supplemental Figure 9.** Sequence annotated map of the pTAMP-DarA-AT recombinant plasmid

**Table S1.** Nucleotide Sequences of the genetic modules used in this study

| NAME | SEQUENCE (5’ 🡪 3’) |
| --- | --- |
| *darA* | atgcataataccttaaatgaaaccgttaaaactcaagaagcactcaattctcttgctgcatcattcaaagagactgaactctcaattactgataaagcactaaacgaattaagcaataaacctaagatccctgagatcacggcctggaactggtcaaaaagcttccaggaaatttaa |
| *darB* | atgaatgctattaacatctcagaatccatgaaacgtttcaatatattggtatcgatactctcagtatcaatagcaatattagcaatttatttactttctactgccttctattcagatgctcgagttgataactattatcaagataatggcaacatttaccgggtggaaaccacctttaatttgcccaatggagaaaaagtgagatcggcaaaatcgccattgccgctcatagacgagctagaaaaagataaaaggataaatcgtgcagactactttttcaaacttaacacgacgattgagttacaaggaaaaaagatcaccaaagttcccgtgtttgcagtcagtcatcaattcctgaataccctgtctccctttaagaaaatcgataatatcctgggagcaaatgagatttacatcacaaaagagttcaataatcgctatctcggcctggaaaatcccaaaggaaaatctattgttctggataatgaaacctatatcatcaaggatatcgtagaaaaacgtcatgattcgagtcttaatatgaatgctattatttcatttaagccaacacttatcaaaaactacaataaagaaatattcaattggtatgatacacatgcttatatttttataaaactctctgatgaaaaatcaatatccaatcgcgatgcgcttcttaatcatattattgcaaccaaagcccccaatcttccaggcgctccatttactgccagtgaatttattcacctatcattaaaaaaatcaaaagatattcattaccaagatggattgcctgatgaaatttctattggtttatcaaaggagataatttatagctcatatatcgcgttcgcatttatcttattttccacattaaccaactattataatttaacttctgctgattcagcagaaaaaagggatatttatcagttaaagaaagcgctaggcgcttcaactttcaatattatttgcgattcactgccggccatgttgatcaaaataatcctttccacccttatctttttgttacttatcataactatatttaatgtctctctctatataaataatatatttaccctattagaagcagacagtttaatatatctagggatattttcagttgttattgcctttatctttatttcatcaatacatttttcattctcattacgtttttatatttatcaaagaaacagaaaaatggatatgagatatgaatcaacccttgcctcttggtttaggaaatcatcattggctattcaattacttgtttccggaatatctatttatttagtcaccggattaaccaatcaatatattcaccttgttgatttacatactccttatgataatgccaagacaatatcaatagcaattaataacgataataataaaaaatcaactattgatgaattaagctatgagtttatcagaaaatacaatacttcaaaaataaccctcagtaattggcgaccatttgatatgtcaagagaaaatatcactattaattattccggccaacaaaacaggaataactatatctcttcaaatgttattactgcggatgaaaatttcactgatgtgtggagaatgaagatattagccggtggtgataaacgagtatatcagagtgataatgctgatgtagtccatgcattagtgacaaaagaatttttaaaacagaatggaattctcaaatatgatgatatttttaacaactactattggtacacaatggaggataaaaagatccaaatcagatttattcagattatagatgatttcaatttaggtgctgtagatgaaccattcaggccaatagttgttttcatcaaaaaagatcatggaaaatatgcttcgctgaatttaaacaatatgaaaaacctttctccggtacttaacgaactatcaaaacagggttttgataataatgaaattgatttaaccagtcacttatttaactcttattatgataattatttaaaagtaataaaattatcatctgccgtcgctattttctcggttctattattagtcatcgcctgctttactaccagcatgactgactttaacgcgatgaaaagggagttaagtataatggaatctattggtggttctatttacaccaatctcattcactttttatcgaaaagtgtcatcccgataatcgcatcaattttcgtggcatatatatcaggaagaactattttagtttatctgcttgatgaatatagtgtgatttacaccgattcttttgtatacagtgctatcgccctatctgctacaatgatattcaccgttattataatgatgactacctatttggtgaattatagaaaattatccgccactaaatttttgtga |
| *darC* | atggatatagaaatcagaaaaaaaggcatacataacagagagaaattactcattctcattttctttatcgcttttatttcatttgttgtatttattatctattatgtcgtttcggctaaagacgctttcgtgaatgaaaatgaagttacccagtttcaggtcagcagcaaagtagatagtgatattctgaaaaccagggcagttgtggttcctgataaaagtgtctctatctcaaccgagatagggggaatcgttaccgatataatgaaaaaaccatcacaagatgttttaaagaatgaagaaatcgtgaaattatctaattttaatttcacattgaataattcttcgatgttagcggacgtgacagataaactcaataatttaataaacataagaataaacttgcaatctgactatcgggatatcaataaccgattcttagaagccgccaaagaactaaaagaagtagaagataaattaaagcgatatcacgatcttgtacataaaaattatatttctaaagaaactatgtccgatttaaggattaaaagagattattggcacgatgtttatctattctacaaaaaattaaaaaatgataaagatcgtgatatatcaaaacagttaaaggaaatcgacgagtttgtcgaaaaacagagaaaactctctgacatcatcgaaaatggtttcgagcagctttcaataaaatcccctatcgccggtaatatcagttcattagatttaattttaggacaacggttaaaacctggcgataaaatagctattgttgatgatttatcaaacttttattttgaatcagaaatcaacgaatactatttaaacaaaataacataccattcatctgccagtttaatctataacaatggaaaaatacctttactggtgaaattaatttcatctgaagtgaataatggaacattcaaagtcagatttgaattggaagataaaaaaacaattaacttcaaacgggggcaatccgttgatgtcatgattaaacttgatgaagatcggaaaatattctccgtaccttcatctatggtgttttctattgacaataaaaactatgtgtttgtttaccatccagataaaaaaattgcccgacgtattcaagtttccaccggccaagataatggttctgatattgagatcaaaagtggtgttactgaaggacaaacgctcgttagctttggtaagaataaattagttaataatgatacagtaaggattgaataa |
| *darD* | atgattagcatgatgaatatctgtaaatcatataaaacgaaattcattcaaacaaatgttttgaatgacataaatctcaatatagataaaggcgaatttatttctataatgggagcgtcagggtcagggaaatcaacattactgaatgtcattggcatgtttgaaacaatcgatagcggtcaattaacactgaacaatagcaatattttaacaatgaaatattctgaaaaaatatcattcagacgagaatttattggttatatttttcaatcattcaatctgttacccaatttgacggtatttgaaaacatagagttaccgttaaaatacagaggttttcctaaaaaacagcggaaaagcaaagtatttgatgcaataaatagttttggattagaaaatcgggaaaatcataaaccgatacaattatctggtggacaacagcaacgtgttgctatcgccagagcaatgatagcaaaccccaccattttattagctgatgaaccaacggggaacctggatagcgtaaatggtcagaatattttatcttcacttaaggagctgaatgaaaacggtacgacgattgttatggtaacccattccgtagaagcggcagcgttttctgacagaatattaacgatgagggatggccacttgcttgtttga |
| *darE* | atggacacaataatccccataaaatatttagattcagacgaatcatcgattcttaagaaatcatctaaaattaactacaggcaattagcttgcagaattatcggtgaaatctccgccgaaaaaatattagatgatgatgaactggctttatataataaagaaatcagtatacatttcagccctgaaattattaatgctaataaattagttgtggttgtgaaagccaccaggctttgcaatttaagatgcacttattgtcactcctgggcagaaggaaaaggaaataccttaacattcttcaatttaatgcgttccattcaccgtttcttatccctaccgaatatcaagcgatttgaattcgtctggcatggcggcgaagtaacgttgttgaatgttaattactttaagaaactcatctggttacaggaacaatttaaaaaaccggatcaagttatcaccaattcggtacagacaaatgccgtcaatattcctgaagattggttagtgttcctcaaaggtattggaatgggggtaggaataagcgttgatggtattccggaaatacacgatagcaggagattagattacagaggaaggccaacatcccataaagtcgcggcaagtatgaaaaagttaagaagttatggcataccttacggtgcgcttatcgtcgtcgaccgcgatgtttatgaatcaaatatagaaaaaatgctctcttatttttacgaaatcggtttaacggatattgaatttctgaatattgtcccagataaccgatgccagccgggtgatgatcctggaggaagttatataacttaccataactatattaatttcctttctaaggttttccgtgtctggtggaatggttatcaaggcaaaatcaatattcgcttgtttgacggatttattgacagtatcaaatcgtcccaaaagaaaatgtcagattgttattgggcgggtaactgttctcaggaaataatcacattagaacctaatggtacggtatcagcatgtgataaatatgttggtgctgaagggaataattatggttcgattattgataatgatcttgggaatttactatctaaatcaaatacaaataaggatcatcttaaagaggaaatggaatcttatgaaaaaatgcatcaatgtaaatggtttcatttgtgtaatggtggatgcccacacgatcgagtgaccaacaggaagcacaatccaaattatgatggttcatgttgtggaaccggcggtttgttggagacaataaaacaaaccatcgcggcgtaa |
| *tlpA_39_* repressor | atgcgtccggcgacatacgaaccagaacagattattgaagcagggctggccctgcaggctgaaggacggaatatcaccgggttcgcactacgtaaccaggtgggtggcggcaatccgacacgtctccgccagatatgggacgaataccaggcttcacagagcacggtcgtcactgaacccgttgccgagctgccagtggaagtggctgaagaagtgaaggccgtctccgccgcgctgtccgaacgcatcacccagctggcgacagaactgaatgacaaggcggtccgggctgcagaacgccgggttgcggaagtcacgcgtgctgccggtgaacagaccgcacaggcagagcgggagctggccgacgccgcgcagacagtcgacgacctggaagaaaaactggttgaactgcaggacagatatgacagtttgacgctggcgctggagtcagaacgttcactgcgtcagcagcatgatgtggagatggcccagctgaaagagcgtcttgcggccgctgaagagaatacccgtcagcgagaggaacggtatcaggagcagaagacagtgctgcaggatgcgcttaatgcggagcaggcacagcacaaaaacacgcgggaagacctgcagaaacgactggagcaaatttctgtcgaagctaatgcgcgtacagaagaactgaagtctgaacgcgataaagtcaatactttccttacccgccttgaatcgcaggaaaatgcgctggcctcagaacgtcagcagcatctggccacccgcgaaacgctgcagcaacgcctcgagcaggccatcgctgacacgcaggcgcgcgccggtgagattgcacttgaacgtgacagagtcagcagcctcaccgcaaggctggaatcgcaggaaaaggcctcctcggagcaactggtgcgtatgggcagtgaaatagccagtctgacagagcgttgcacacagctggaaaaccagcgtgatgatgcccgtctggagacgatgggggagaaagaaacggtcgcggcactgcgtggtgaggctgaagccctgaagcgtcagaaccagtcactgatggcggcgctttcaggcaataaacagaccggtggccagaatgcgtga |
| *txe*  toxin | atgattaaggcttggtctgatgatgcttgggatgattatctttattggcatgagcaaggaaacaaaagcaatataaaaaagattaacaagttaataaaagatatcgatcgttccccctttgctggattaggaaaacctgagccattaaagcatgatttatctggaaaatggtccagaagaattacagatgaacatagactgatatatagagttgaaaatgaaacgatatttatttattctgcaaaagatcactattaa |
| *axe*  antitoxin | atggaagcagtagcttattcaaatttccgccaaaatttacgtagttatatgaaacaagttaatgaggatgctgaaacacttattgtaacaagtaaagatgtagaagatacagttgttgtattatcaaaaagagattatgattctatgcaagaaacgttgagaacactttctaataattacgtcatggaaaaaattcgtcgaggagatgaacaattctccaaaggtgcatttaaaacacatgacttaatcgaggttgaatctgatgattaa |
| *t7*  *RNAP* | atgaacacgattaacatcgctaagaacgacttctctgacatcgaactggctgctatcccgttcaacactctggctgaccattacggtgagcgtttagctcgcgaacagttggcccttgagcatgagtcttacgagatgggtgaagcacgcttccgcaagatgtttgagcgtcaacttaaagctggtgaggttgcggataacgctgccgccaagcctctcatcactaccctactccctaagatgattgcacgcatcaacgactggtttgaggaagtgaaagctaagcgcggcaagcgcccgacagccttccagttcctgcaagaaatcaagccggaagccgtagcgtacatcaccattaagaccactctggcttgcctaaccagtgctgacaatacaaccgttcaggctgtagcaagcgcaatcggtcgggccattgaggacgaggctcgcttcggtcgtatccgtgaccttgaagctaagcacttcaagaaaaacgttgaggaacaactcaacaagcgcgtagggcacgtctacaagaaagcatttatgcaagttgtcgaggctgacatgctctctaagggtctactcggtggcgaggcgtggtcttcgtggcataaggaagactctattcatgtaggagtacgctgcatcgagatgctcattgagtcaaccggaatggttagcttacaccgccaaaatgctggcgtagtaggtcaagactctgagactatcgaactcgcacctgaatacgctgaggctatcgcaacccgtgcaggtgcgctggctggcatctctccgatgttccaaccttgcgtagttcctcctaagccgtggactggcattactggtggtggctattgggctaacggtcgtcgtcctctggcgctggtgcgtactcacagtaagaaagcactgatgcgctacgaagacgtttacatgcctgaggtgtacaaagcgattaacattgcgcaaaacaccgcatggaaaatcaacaagaaagtcctagcggtcgccaacgtaatcaccaagtggaagcattgtccggtcgaggacatccctgcgattgagcgtgaagaactcccgatgaaaccggaagacatcgacatgaatcctgaggctctcaccgcgtggaaacgtgctgccgctgctgtgtaccgcaaggacaaggctcgcaagtctcgccgtatcagccttgagttcatgcttgagcaagccaataagtttgctaaccataaggccatctggttcccttacaacatggactggcgcggtcgtgtttacgctgtgtcaatgttcaacccgcaaggtaacgatatgaccaaaggactgcttacgctggcgaaaggtaaaccaatcggtaaggaaggttactactggctgaaaatccacggtgcaaactgtgcgggtgtcgataaggttccgttccctgagcgcatcaagttcattgaggaaaaccacgagaacatcatggcttgcgctaagtctccactggagaacacttggtgggctgagcaagattctccgttctgcttccttgcgttctgctttgagtacgctggggtacagcaccacggcctgagctataactgctcccttccgctggcgtttgacgggtcttgctctggcatccagcacttctccgcgatgctccgagatgaggtaggtggtcgcgcggttaacttgcttcctagtgaaaccgttcaggacatctacgggattgttgctaagaaagtcaacgagattctacaagcagacgcaatcaatgggaccgataacgaagtagttaccgtgaccgatgagaacactggtgaaatctctgagaaagtcaagctgggcactaaggcactggctggtcaatggctggcttacggtgttactcgcagtgtgactaagcgttcagtcatgacgctggcttacgggtccaaagagttcggcttccgtcaacaagtgctggaagataccattcagccagctattgattccggcaagggtctgatgttcactcagccgaatcaggctgctggatacatggctaagctgatttgggaatctgtgagcgtgacggtggtagctgcggttgaagcaatgaactggcttaagtctgctgctaagctgctggctgctgaggtcaaagataagaagactggagagattcttcgcaagcgttgcgctgtgcattgggtaactcctgatggtttccctgtgtggcaggaatacaagaagcctattcagacgcgcttgaacctgatgttcctcggtcagttccgcttacagcctaccattaacaccaacaaagatagcgagattgatgcacacaaacaggagtctggtatcgctcctaactttgtacacagccaagacggtagccaccttcgtaagactgtagtgtgggcacacgagaagtacggaatcgaatcttttgcactgattcacgactccttcggtaccattccggctgacgctgcgaacctgttcaaagcagtgcgcgaaactatggttgacacatatgagtcttgtgatgtactggctgatttctacgaccagttcgctgaccagttgcacgagtctcaattggacaaaatgccagcacttccggctaaaggtaacttgaacctccgtgacatcttagagtcggacttcgcgttcgcgtaa |
| alanine racemase (*alr*) | atgcaagcggcaactgttgtgattaaccgccgcgctctgcgacacaacctgcaacgtcttcgtgaactggcccctgccagtaaaatggttgcggtggtgaaagcgaacgcttatggtcacggtcttcttgagaccgcgcgaacgctccccgatgctgacgcctttggcgtagcccgtctcgaagaagctctgcgactgcgtgcggggggaatcaccaaacctgtactgttactcgaaggcttttttgatgccagagatctgccgacgatttctgcgcaacattttcataccgccgtgcataacgaagaacagctggctgcgctggaagaggctagcctggacgagccggttaccgtctggatgaaactcgataccggtatgcaccgtctgggcgtaaggccggaacaggctgaggcgttttatcatcgcctgacccagtgcaaaaacgttcgtcagccggtgaatatcgtcagccattttgcgcgcgcggatgaaccaaaatgtggcgcaaccgagaaacaactcgctatctttaataccttttgcgaaggcaaacctggtcaacgttccattgccgcgtcgggtggcattctgctgtggccacagtcgcattttgactgggtgcgcccgggcatcattctttatggcgtctcgccgctggaagatcgctccaccggtgccgattttggctgtcagccagtgatgtcactaacctccagcctgattgccgtgcgtgagcataaagccggagagcctgttggttatggtggaacctgggtaagcgaacgtgatacccgtcttggcgtagtcgcgatgggctatggcgatggttatccgcgcgccgcgccgtccggtacgccagtgctggtgaacggtcgcgaagtaccgattgtcgggcgcgtggcgatggatatgatctgcgtagacttaggtccacaggcgcaggacaaagccggggatccggtcattttatggggcgaaggtttgcccgtagaacgtatcgctgaaatgacgaaagtaagcgcttacgaacttattacgcgcctgacttcaagggtcgcgatgaaatacgtggat |

**Table S2.** List of primers and their corresponding sequences used for qRT-PCR and qPCR analysis

| **Primer Name** | **Sequence (5’🡪 3’)** | **Description** | **Reference** |
| --- | --- | --- | --- |
| **darA Fwd** | GCACTCAATTCTCTTGCTGCAT | qRT-PCR forward primer for darA | This study |
| **darA Rev** | TTTTGACCAGTTCCAGGCCG | qRT-PCR reverse primer for darA | This study |
| **T7RNAP Fwd** | CGAGAACATCATGGCTTGCG | qRT-PCR forward primer for T7RNAP | This study |
| **T7RNAP Rev** | CCCAGCGTACTCAAAGCAGA | qRT-PCR reverse primer for T7RNAP | This study |
| **pUC Fwd** | TTGCCGGATCAAGAGCTACC | qPCR forward primer for pUC origin | This study |
| **pUC Rev** | GGCGGTGCTACAGAGTTCTT | qPCR reverse primer for pUC origin | This study |
| **16S rRNA Fwd** | CATGCCGCGTGTATGAAGAA | qRT-PCR forward primer for 16S rRNA | Smati et al., 2013 |
| **16S rRNA Rev** | CGGGTAACGTCAATGAGCAAA | qRT-PCR reverse primer for 16S rRNA | Smati et al., 2013 |
